# Comparison of diagnostic methods for assessment of *Ostertagia ostertagi* exposure in Norwegian dairy herds

**DOI:** 10.1186/s13028-023-00712-y

**Published:** 2023-11-29

**Authors:** Tonje Opsal, Ingrid Toftaker, Lucy Robertson, Ian Woolsey, Lisbeth Hektoen

**Affiliations:** 1 Department of Production Animal Clinical Sciences, Faculty of Veterinary Medicine, Norwegian University of Life Sciences, Universitetstunet 3, Ås, 1433 Norway; 2 Department of Paraclinical Sciences, Faculty of Veterinary Medicine, Norwegian University of Life Sciences, Universitetstunet 3, Ås, 1433 Norway

**Keywords:** Antibody level, Bulk tank milk, ELISA, Gastrointestinal nematodes, Optical density rate, Pasture parasites, Svanovir

## Abstract

**Background:**

The gastrointestinal nematode (GIN) *Ostertagia ostertagi* can cause severe disease in first season grazers (FSG) and impaired performance due to subclinical infections in adult cows. Diagnostic methods to assess exposure include faecal egg count and detection of specific antibodies using antibody-ELISAs resulting in an optical density ratio (ODR). Using the ELISA test on bulk tank milk (BTM) allows for a herd level diagnosis. Appropriate use of diagnostic methods for evaluation of *O. ostertagi* exposure is required to optimize herd parasite surveillance and aid in a sustainable control regime. The aim of this study was to describe the relationship between different diagnostic tests used to assess GIN exposure in Norwegian production systems. A cross-sectional field study was carried out in twenty herds in Norway in the fall of 2020. Serum and faecal samples were taken from 380 individuals, of which 181 were FSG and 199 were cows. In addition, milk was collected from every cow and one BTM sample was taken from each herd. Faecal egg counts were performed. The distribution of ODR values in individual samples within and between herds and the associations between BTM ODR and individual ODR values were described. The data were analysed using visual assessment of scatter plots, Pearson correlation coefficients and linear regression.

**Results:**

A high variability of the within-herd individual ODR values in serum and milk in every herd was detected. The ODR in BTM explained a low degree of the variation in the individual serum and milk samples. When plotting the ODR results in milk or serum according to four BTM categories, the distribution of ODR values were notably different in the highest and lowest BTM categories. The correlation between individual milk and serum samples was moderate (r = 0.68), while the highest correlation (r = 0.81) was between the BTM ODR and the group average individual milk samples.

**Conclusions:**

A poor predictive ability for BTM ODR to assess individual ODR values in both FSG and cows was demonstrated. However, the study indicates that the evaluation by ELISA test on BTM to assess exposure to GIN could be useful in herds with a very high or low BTM ODR.

## Background

The gastrointestinal nematode (GIN) *Ostertagia ostertagi* is among the most important parasites contributing to bovine parasitic gastroenteritis in temperate and subtropical regions [1]. The developing larvae destroy the glandular tissue in the abomasum compromising digestion [2]. Severe disease can occur in first season grazers (FSG), whereas in adult animals, subclinical infections associated with economic losses due to impaired performance including reduced milk yield are common [3–6]. Treatment with anthelmintics has been extensively used to control parasite infection, however, as reviewed by Rose et al. [7], an evolving anthelmintic resistance has been detected in several countries. Due to strict regulations concerning food safety and ecotoxicity concerns, the development of new anthelmintic products is not considered to keep pace [8]. To optimize herd parasite surveillance and target treatment to reduce unnecessary use of anthelmintics, knowledge of appropriate and correct use of diagnostic methods is required [9, 10].

Ostertagiosis can be diagnosed by faecal egg counts (FEC) of nematode eggs and reported in eggs per gram (EPG), determination of serum pepsinogen levels, or by measuring parasite-specific serum antibody levels [11]. Molecular methods, such as qPCR, ddPCR, automated PCR platforms and DNA sequencing technologies, are more recent methods for detection and quantification, as well as detailed studies into GIN species diversity [12–15]. The use of FEC is the most widely used diagnostic technique for monitoring infection patterns in FSG as it is non-invasive, relatively cost-effective and does not require sophisticated laboratory equipment [16]. However, it correlates poorly with worm burden and subclinical production losses [17]. The relationship between FEC and worm burden may only be consistent until 2 months after onset of the pasture period. After that time period, the method loses diagnostic value as the acquired immunity restricts the fecundity of established adult worms [18, 19]. Performing FEC is still applicable to estimate pasture contamination with parasite eggs and to monitor the efficacy of anthelmintic treatment by interpretation of a FEC reduction test [8, 20, 21]. Previous exposure to *O. ostertagi* can be assessed by measuring serum pepsinogen levels, which increase due to release of accumulated pepsinogen into the blood stream as a sequela to abomasal worm activity [22]. A rising serum pepsinogen level has shown significant correlation with *O. ostertagi* adult-worm burden at slaughter [23], but the titre decreases rapidly in the absence of continuous exposure to the abomasal worm. Conversely, the antibody level may further increase after housing, due to the lag phase between infection and the appearance of antibodies [11]. An enzyme-linked immunosorbent assay (ELISA) using a crude adult *O. ostertagi* antigen can be used to detect antibodies against *O. ostertagi* in serum and milk. The results are expressed as optical density ratios (ODR). The antibody level has shown a weak correlation with parasite loads [24] and reflects the level of previous exposure rather than active infection [9]. On an individual level, associations with lactation number, days in milk (DIM) and milk yield have been described [25–27]. Furthermore, the specificity of the *O. ostertagi* antibody ELISA test is affected by cross reactivity with antibodies raised against other helminths such as *Cooperia oncophora* [28] and *Fasciola hepatica* [17]. For adult cows, both individual milk (IM) samples as well as bulk tank milk (BTM) samples can be tested by ELISA. The use of BTM testing instead of individual samples to assess exposure has both practical and economic benefits, as BTM sampling is easy, non-invasive, and often routinely performed. However, the choice of sampling schemes and the antibody titre being affected by the number and relative seropositivity of contributors are among many factors to consider when interpreting the ODR in BTM [29]. Animals not producing milk do not contribute to the sample, which may be a factor to consider when evaluating the results of a BTM test. Nonetheless, studies have shown a significant positive association between herd exposure to pasture and increased OD-values in the BTM [6, 30–32], even when considering heifers [3]. This encourages the use of BTM ELISA as a herd-level test at the end of the pasture season. Moreover, vast regional differences in exposure level have been detected in the northwestern European dairy herds, where the BTM ODR were associated with pasture management and climatic factors [32, 33]. Screening of BTM to identify high infection clusters could thus be used to inform monitoring and control programs in these regions. Additionally, calculations of economic loss due to GIN infections have been documented [9, 34–37] and may encourage farmers to perform diagnostic testing in their herds [15]. A conversion chart indicating milk yield loss when BTM ODR > 0.5 has been developed [38, 39]. This threshold is only validated for some European countries, and it has been emphasised that regional epidemiological surveys are required to validate diagnostic assessments that are applicable in different production systems [38].

Current knowledge about pasture parasites in Norway is limited. No national prevalence studies of *O. ostertagi* have been performed and the availability of commercial diagnostic tests is scarce. Norwegian cattle herds are relatively small, with a mean herd-size of 29.3 cow-years in 2020 [40]. The pasture season for Norwegian dairy cattle, occurring from May to October, has an average duration of 4.3 months [41], which is short compared to the average grazing season duration in other northwestern European countries [33]. Prior to implementing new methods for quantification of GIN infections, there is a need to investigate how different diagnostic tests can assess GIN exposure in Norwegian production systems. This is an important step before these can be established as a herd-health management tool to aid in the identification of which cows, herds or regions would benefit from improvements in pasture parasite management. The aims of this study were: (1) to describe the correlation between ODR values in milk and serum samples using a *O. ostertagi* antibody ELISA and to describe the distribution of ODR values within and between herds, (2) to investigate associations between BTM ODR and individual ODR values and (3) to evaluate the FEC in samples from these herds.

## Methods

### Study population

A cross-sectional field study was carried out in 20 herds in Norway in the autumn of 2020. Herd visits were conducted from August 30th to November 2nd 2020. Each herd was visited once. The herds were located in the eastern counties of Oslo (*n* = 1), Viken (*n* = 12), and Telemark (*n* = 4), as well as in the county Rogaland (*n* = 3) on the southwest coast of Norway. Farmers were recruited between March and September of 2020, primarily through acquaintance with project participants and via webpages targeting both dairy farmers and local veterinarians. Purposive convenience sampling was used to select herds in which exposure to parasites was expected based on information provided by the farmers. All included herds were members of the Norwegian Dairy Herd Recording System (NDHRS), allowing extraction of health and production data from farm records. The animals sampled had all been on pasture during the previous grazing season, which, according to Norwegian animal welfare legislation, is a requirement for all female cattle older than 6 months. The FSG in the study were heifers between 9 months and 2 years of age. They mostly grazed on home pastures that had been used for FSG every year, with no systematic approach to rotational grazing. Farmers that had treated with anthelmintics in 2020 were excluded. The farmers were provided with an information letter describing the use of data for research and publication and agreed to sampling and sharing of farm records.

The selected herds were all visited shortly (1–21 days) after housing. The sampling was done by the first author, or by local veterinarians following a written protocol provided by the project. At each herd visit, blood samples from the tail vein and IM samples were taken from a convenience sample of 10 lactating cows, including cows of different ages and lactation stages, as far as possible. Additionally, blood samples were taken from preferably 10, but at least 5 FSG. The number of samples was maximised within the constrains in terms of time and budget. Faecal samples were collected with a gloved hand from the rectum from all individuals. The plastic glove was immediately inverted and tied allowing storage of the faecal sample in the glove used for collection. One BTM sample was taken in each herd. The milk and blood samples were collected by the milk truck driver at the ordinary pick-up of milk and cooled at a temperature of 2–4 °C until freezing at the laboratory (TINE Laboratory, Molde, Norway). The blood samples were centrifuged to obtain serum, and milk and serum samples were subsequently stored at − 20 °C for up to 5 months before analysis. The faecal samples were brought to the Parasitology Laboratory at the Faculty of Veterinary Medicine at the Norwegian University of Life Sciences immediately after collection, or for faecal samples collected by local veterinarians, shipped to the laboratory by overnight express mail.

The collection of samples for this study was considered a non-experimental clinical procedures and did not require ethical approval in Norway.

### Laboratory analyses of faeces, blood and milk

Egg counts in 3 g of faeces, recorded as EPG, were determined using a modified McMaster method [42, 43]. Levecke et al. [44] investigated the analytical properties of a modified McMaster technique similar to the one used in the present study and reports an analytical sensitivity of 10–50 EPG. Two of the project participants were responsible for the FEC, which was performed within 4 days after sampling. After analysis, the faecal samples were stored in vacuum sealed plastic bags. The analyses of serum and milk were carried out at TINE Mastitis Laboratory in Molde. Specific antibodies against *O. ostertagi* in BTM, IM, and individual serum (IS) samples were detected using the SVANOVIR® *Ostertagia-Ab* ELISA kit (Svanova Biotech, Uppsala, Sweden), which is a semi-quantitative test based on a crude adult-worm capture antigen [45]. The test is commercially available and marketed for use on BTM samples, however, it has been used for serum and milk samples [4, 46, 47] and a recent review confirmed its suitability to assess past GIN exposure in FSGs after housing [48]. The IM samples were used undiluted, and the IS samples were diluted 1:140 before being tested. Results from all tests were expressed as ODRs, which were calculated following the formula ODR=(OD / NC)/(PC /NC), where NC and PC are the OD values of the negative control and positive control, respectively.

### Production data

To collect information about the herds from a period relevant to the time of sampling, we chose to extract data from the NDHRS for a study period from May 1st 2020 to April 31st 2021. The retrieved herd level variables were: herd size (cow-years, where one cow-year equals 365 days for a cow in a herd, calculated for each cow from date of first calving) and group average milk yield (kg energy corrected milk (ECM)/cow/day). Individual level variables were: lactation number, days in milk and individual milk yield (kg ECM/day) of the included individuals. Herd size was defined as the herds’ mean number of cow-years in the study period. For DIM, values above 600 days were deemed unlikely and removed. Mean individual milk yields were calculated based on the milk production of the cow given at 6–12 monthly test days, measured as kg ECM/day. One mean milk yield value of 3 kg ECM/day was deemed unlikely and removed.

The EPG, ODR values, and production data were received as Excel files, Excel Office 365 (Microsoft Inc) and exported to STATA 16 [49] for data management and statistical analysis.

### Statistical analysis

A descriptive table was made for production data and herd demographics. The medians, means, standard deviations and ranges of the ODR and FEC values were calculated for the individual values as well as the herd level averages. Histograms and Shapiro-Wilk test were used to assess the data for normal distribution. The FEC were log transformed to reduce the impact of the few high values.

A graph matrix of scatter plots was made to visually assess the correlation between ODR values of IM and IS, as well as the correlation between IS and IM ODR values and BTM. A scatter plot with a lowess-smoothed curve was generated to visualize the association between BTM ODR and IM ODR. Pearson correlation coefficients were calculated between log EPG and IS ODR, log EPG and IM ODR, IS ODR and IM ODR, the group averages of IS ODR and IM ODR, and the group average ODR values and BTM ODR. The mean BTM ODR in the study sample was applied as a cut-off to divide the herds into high and low BTM ODR-categories. For the resulting 2 categories, a t-test was performed to evaluate the statistical significance when comparing the means of the IM ODR values in the high and low BTM ODR herds. Furthermore, the herds were divided into four categories depending on the BTM ODR result. The following cut-points were used to define categories of equal intervals: 0.20–0.40, 0.41–0.60, 0.61–0.80 and 0.81–1.0. The distributions of the IM and IS ODR values of the individuals in the four BTM categories were illustrated using 2 box plots. The associations between ODR in individual milk/serum samples and individual milk yield (average ECM kg/day), DIM and lactation number were visually assessed using scatter plots (results not shown). The ability of a BTM test to predict the IS ODR values was explored using a mixed linear regression model with BTM ODR, DIM and group (FSG, first lactation, second lactation, third and higher lactation) as the candidates for explanatory variables and IS ODR as the response variable. A herd random effect was included to account for dependence between cows from the same herd. The model was fit using a manual backward stepwise elimination procedure, with a selection threshold of *P* < 0.05. The final model, after elimination, included BTM ODR and group together with the random effect of herd and can be written as:

$$Yij = {\beta _0} + {\beta _1}{X^{BTM}}j{\text{ }} + {\beta _2}{X^{group}}_{ij} + {\upsilon _j} + {\varepsilon _{ij}},$$

where subscripts *i* and *j* denote the *i*th individual of the *j*th herd, respectively *Yij* = individual serum ODR, X^BTM^ is the BTM ODR variable, X^group^ is the group variable, υ_j_ as the herd random effect and ${\epsilon }_{i }$are identically and independently distributed $N(0,{\sigma }^{2})$ error terms.

The intraclass correlation coefficient was calculated. All models were checked for residual normality and homoscedasticity by visually inspecting plots of residuals against fitted values and quantile-quantile plots of residuals.

## Results

The results from 13 samples were missing at the time of analysis. 6 IS and 5 IM samples lacked the *O. ostertagi*-Ab ELISA test result, and 2 faecal samples were not returned to the lab.

## Herd and individual data

Of the 20 herds, 19 were conventional and one was organic. The mean herd size during the study period was 33.4 cow years. Of 380 included individuals, 181 were FSG and 199 were lactating cows. The distribution of included individuals from every herd was a mean of 9.95 cows (range 9–10) and a mean of 9.4 FSG (range 5–10). Production data from 19 of the 20 herds were provided. The average herd milk yield was 25.6 kg ECM/cow/day (Table 1). Of the 191 cows for which production data was provided, 82 (43%) were in their 1st lactation, and 109 (57%) were in second lactation or higher. The average individual milk yield in the study period, calculated for 181 individual cows, was 24.3 kg ECM/day.

**Table 1:** Production data of 20 herds included in a field study in autumn 2021. These data were retrieved from the Norwegian dairy herd recording system from May 1st 2020 till April 31st 2021

	Study sample			Norwegian dairy units
	Median	Mean	Range	Mean
Herd size (cow years)	34.7	33.4	10.4–87.9	29.4¹
Milk yield (kg ECM/day/cow)	25.9	25.6	17.8–33.2	23.6¹
Days in milk	145	154.2	0–567	N/A

ECM: Energy corrected milk N/A: Not applicable ¹Data gathered from “Statistikksamling fra Husdyrkontrollen og Helsekortordningen. TINE SA” [40]

### Relationship between antibody levels in BTM and individual samples

**Fig. 1:**
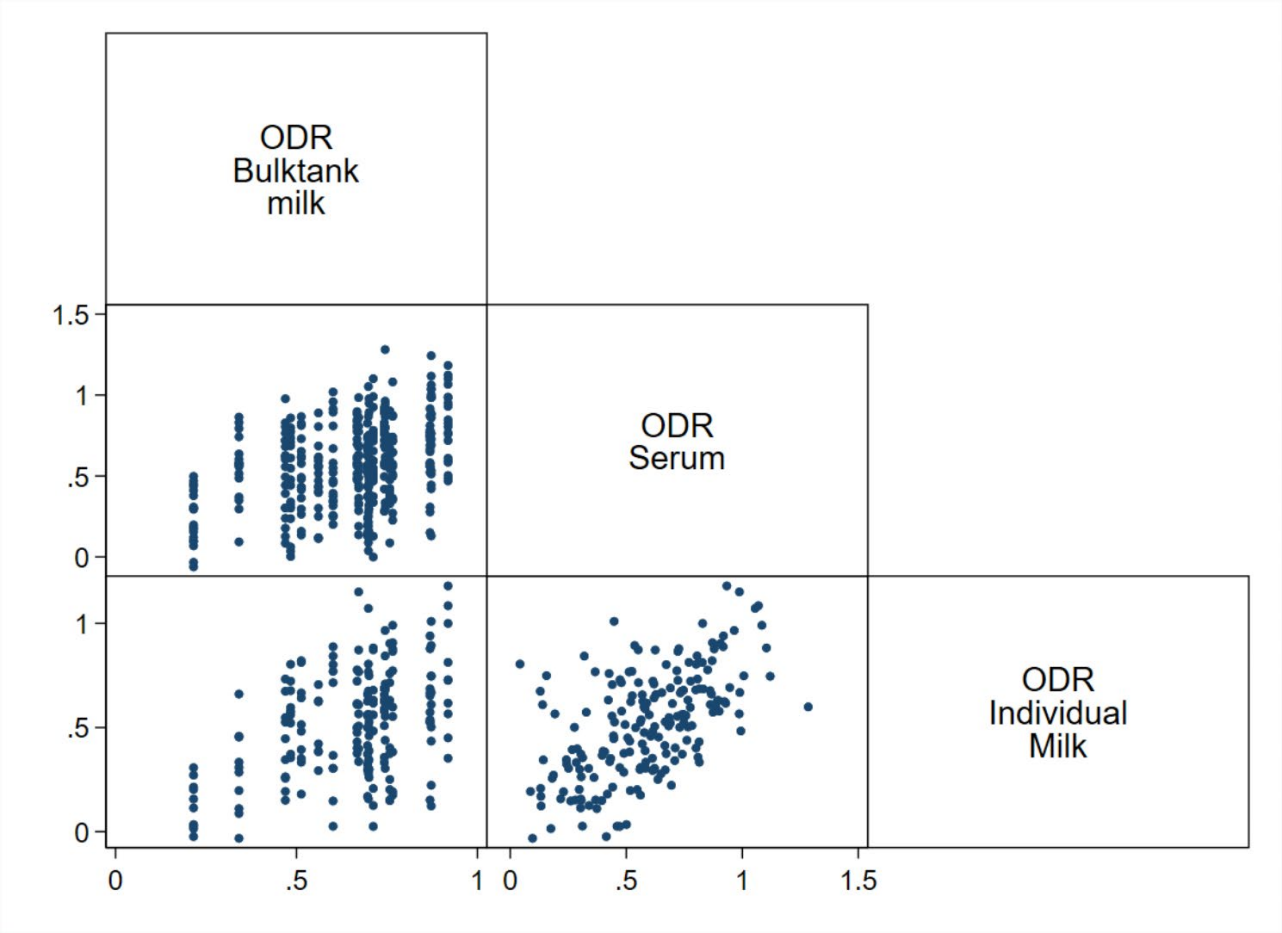
Graph matrix showing the relationship of the ODR values using an indirect antibody ELISA for *Ostertagia ostertagi* test on bulk tank milk, individual milk (*n* = 194) and cow serum samples (*n* = 197) from 20 herds in Norway

**Fig. 2:**
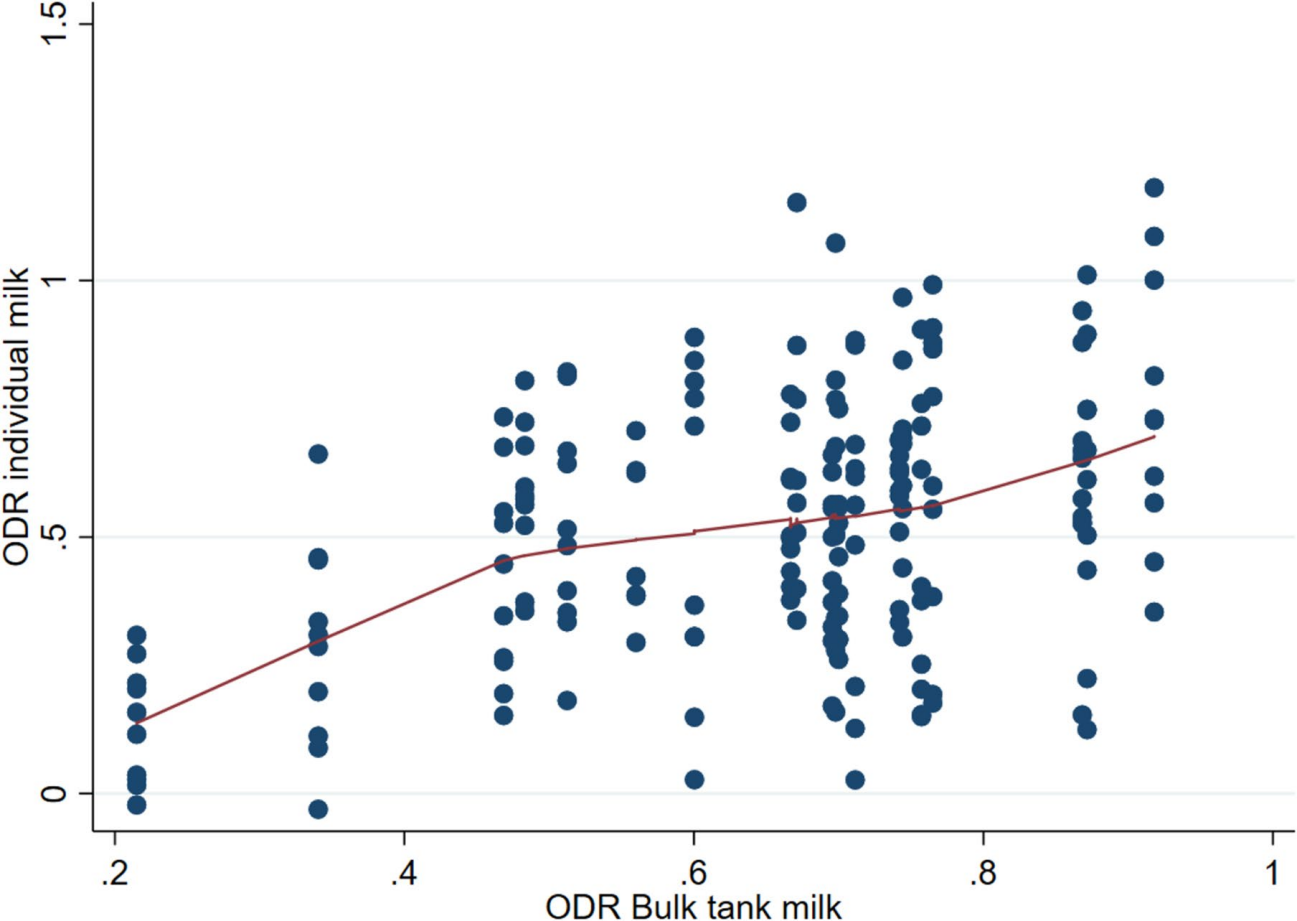
Scatterplot of bulk tank milk ODR values versus individual milk ODR values with a lowess fitted line using an indirect antibody ELISA for *Ostertagia ostertagi* in 194 cattle from 20 herds in Norway

**Fig. 3:**
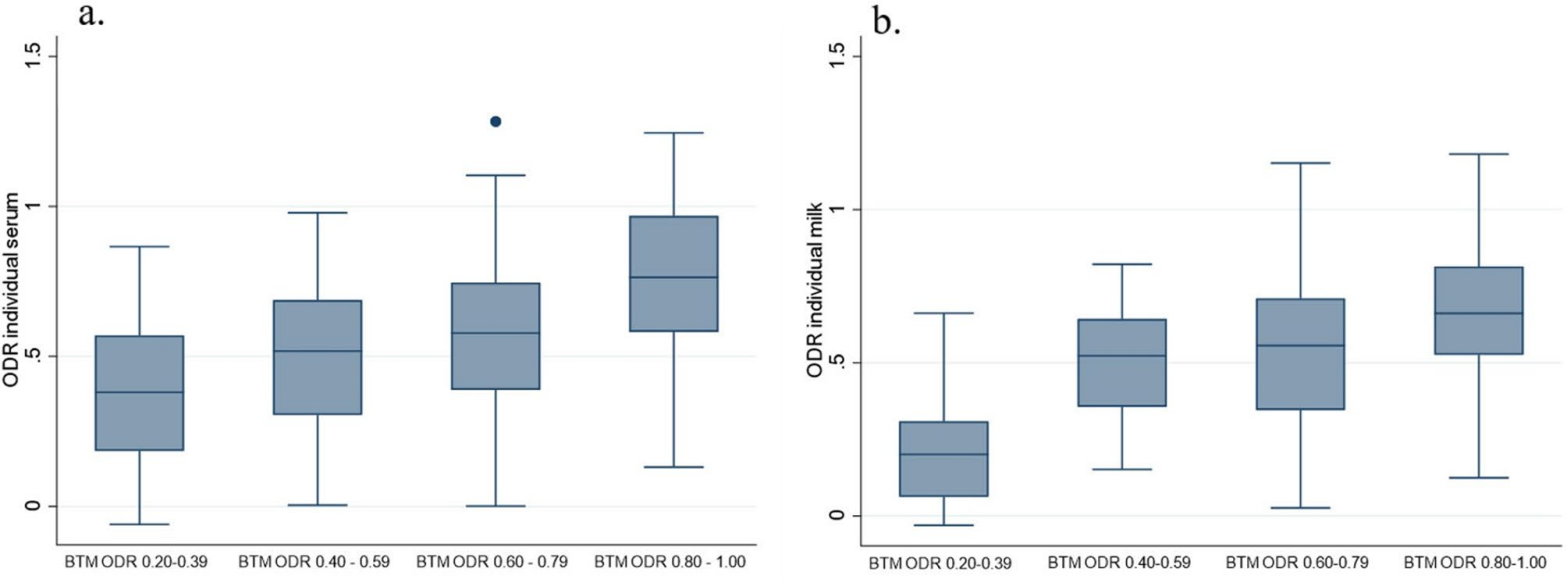
Distribution of **(a)** individual serum (*n* = 374) and **(b)** individual milk (*n* = 194) ODR values according to four categories of bulk tank milk (BTM) using an indirect antibody ELISA for *Ostertagia ostertagi* in 20 herds in Norway

The mean BTM ODR value for *O. ostertagi* of the 20 herds in the study was 0.65, with a range of 0.22–0.92 (Table 2). A wide variation in the individual ODR values was detected in all study herds, as illustrated in Figs. 1 and 2, and 3. The t-test showed a significant difference in IM ODR between the low and high BTM ODR categories (*t*(192) = 16.63, P < 0.001) with a mean difference in IM ODR values between groups of 0.42, 95% CI [0.37,0.47]. By visual evaluation of the IM and IS ODR values according to the four BTM categories (Fig. 3), the distribution was notably different in the highest and lowest BTM category. Nevertheless, there were large within-herd variations resulting in cows with high ODR values in the lowest BTM-category (ODR: 0.20–0.39) and cows with low ODR values in the highest bulk tank category (ODR: 0.80–1.00). The correlation between group average IS ODR in FSG and BTM ODR (Table 3) was moderate (r = 0.63), while a higher correlation was observed between the group average IM ODR samples and BTM ODR (r = 0.81). The BTM ODR was higher than the group average IM ODR in 90% (18/20) of the samples, with an average increase of 25% in BTM ODR compared to group average IM ODR.

**Table 2:** Descriptive statistics of faecal egg counts (FEC) of strongyle eggs and ELISA tests using the SVANOVIR® *Ostertagia-Ab* ELISA kit

Sample material	Sampling unit	Unit	N	Mean	Median	SD	Range
Faeces	FSG	EPG	180	97.11	20	220.31	0–1750
	FSG	Log EPG	180	2.68	3.00	2.22	0 -7.47
Serum	FSG	ODR	177	0.55	0.57	0.28	-0.06 -1.24
	Cows	ODR	197	0.58	0.58	0.24	0.04–1.28
Milk	Cows	ODR	194	0.52	0.53	0.25	-0.03–1.18
Bulk tank milk	Herd	ODR	20	0.64	0.70	0.18	0.22–0.92

FSG: First season grazers EPG: Eggs per gram ODR: Optical density ratio SD: Standard deviation

**Table 3:** Pearson correlation coefficients (r) of pairwise comparisons between ODR values in milk, serum and bulk tank milk on individual and group average levels from 20 Norwegian dairy herds

	*n*	r
Individual ODR cow serum x Individual ODR milk	192	0.58
Group average ODR cow serum x group average ODR milk	20	0.73
Group average ODR FSG serum x ODR BTM	20	0.63
Group average ODR cow serum x ODR BTM	20	0.69
Group average ODR cow milk x ODR BTM	20	0.81

BTM: Bulk tank milk FSG: First season grazers ODR: Optical density ratio

Results from the mixed effects linear regression model using all the serum ODR values are shown in Table 4. There was a small positive association between BTM ODR and the IS ODR (*p* < 0.001). Cows in their 1st lactation had higher estimated antibody levels than FSG. The intraclass correlation coefficient was 0.07, reflecting that only 7% of the variation in ODR from IS samples was explained by herd as a random effect. No major shortcomings of the model were detected through assessment of the residual plots. Based on visual assessment of scatter plots there were no indications of an association between individual ODR values and individual milk yield. In order to conduct a more comprehensive analysis of this relationship, it would have been imperative to gather supplementary data. However, this task was beyond the scope of this study.

**Table 4:** Results of the mixed linear regression model to estimate the association between the bulk tank ODR and individual ODR values from serum samples of 374 cows and first season grazers

Variables			Coefficient	95% CI	Std.error	*p*-value	*n*
Bulk tank ODR			0.59	(0.37–0.80)	0.11	< 0.001	20
Group							
	First season grazers		Baseline				188
	First lactation		0.10	(0.04–0.16)	3.24	0.001	82
	Second lactation		0.01	(-0.06–0.08)	0.27	0.784	57
	≥ 3 lactations		-0.07	(-0.14–0.01)	-1.79	0.073	52
Constant			0.18	(0.03–0.32)	0.07	0.016	
Random effect variance							
	Herd		0.00	(0.00–0.01)	0.00		

CI: Confidence interval ODR: Optical density ratio

### Relationship between individual samples of serum, milk, and faeces

The individual log transformed EPGs had a very low correlation with both IM ODR in cows (r = 0.03) and IS ODR in both FSG (r = 0.11) and cows (r = 0.01). The group average IS ODR was higher than the group average IM ODR in 70% (14/20) of the herds. The correlation between the IS ODR and IM ODR, visually assessed in Fig. 1, was moderate (r = 0.58; Table 3). The correlation was higher in low-yielding cows with an average milk yield of < 25 kg ECM/day (r = 0.61) versus in high-yielding cows with an average milk yield of > 25 kg ECM/day (r = 0.56).

## Discussion

There was a large within-herd variation in ODR values for both IS and IM samples. The Pearson correlation coefficient in this study was highest between the BTM ODR and the group average IM ODR, while a moderate correlation was detected between the individual ODR values in IS and IM samples. The evaluation of the regression model further demonstrated that BTM explained a low degree of the variation in individual ODR values in both FSG and adult cows. Nevertheless, the distribution of individual ODR levels was clearly different when comparing the herds close to the minimum and maximum BTM ODR values. Due to relatively few study herds being included, the possible inferences that can be made from this are limited. However, these data indicate that BTM testing might be of value to identify herds or regions with a substantial exposure to *O. ostertagi* or to single out herds where the level of exposure does not appear to be of concern.

The farms included in this study had a mean BTM ODR value of 0.65, which is comparable to the results in other European surveys where cows were pastured [33, 38]. In a survey of 5 countries located in northwestern Europe in 2010, which also used the SVANOVIR® *Ostertagia*-Ab ELISA kit, an average of 0.66 ODR in BTM on country-level was classified as “intermediate” when considering exposure to GIN [33]. Thus, a moderate exposure is indicated in our study sample based on the ODR levels in their BTM.

The high correlation detected between the BTM ODR and the group average IM samples (r = 0.81), indicates that BTM ODR may estimate the average level of GIN exposure in cows. This correlation was found to be moderate in other studies, with coefficients of r = 0.45 and r = 0.54 [30, 50], while a correlation of r = 0.72 was detected using the mean of two IM samples collected from all lactating animals two months apart [25]. The higher correlation obtained in our study might be related to the small size of Norwegian herds. The sampled cows in our study were a relatively large proportion of the cows contributing to the bulk tank, thus the difference between the mean ODR of collected IM samples and the ODR from a pooled sample of all lactating cows (i.e., BTM) could be smaller than for studies with larger herds. The higher BTM ODR relative to the group average IM ODR is similar to results from previous studies. A consistently higher BTM ODR than the group average IM ODR has been described [25], while other studies have reported that the BTM ODR was 22% [30] and 53% [51] higher than the average IM ODR. These findings have been attributed to the plateau phase in the ELISA test reaction; as the antibody level in the sample exceeds a certain antibody concentration, a recognized non-linear relationship develops between antibody concentration and the ELISA test result [25].

The moderate correlation between the ODR values in IS and IM corroborates results from other studies, ranging from r = 0.45 [52] to r = 0.53 [30]. The relationship between serum and milk can be complex. Antibodies appear earlier in serum than in milk, and the concentration of antibodies in serum has previously been shown to be approximately 30 times greater than in milk [53]. Additionally, a dilution effect in high-yielding cows was suggested by Sanchez et al. (2002) [30]. Our results support this suggestion, as the correlation between IM and IS ODR was reduced in high-yielding cows compared to low-yielding cows.

As expected from the large within-herd variation, the regression model showed poor predictive ability for BTM ODR to assess individual ODR values in both FSG and cows. The majority of the variation in individual ODR values was found between cows, while only 7% is explained by a herd effect. The significant effect of lactation group in this study has also been found in other studies [25, 27, 30], and may reflect higher levels of acquired immunity in older cows [27]. However, evaluations of lactation number and other cow factors (DIM, somatic cell count) in relation to ODR in individual samples have provided differing results and the effects of these variables are often reported as low [25–27]. This implies that additional factors are important in explaining individual variation in antibody response in animals grazing the same pasture. Genetic traits have been significantly associated with the ability of dairy cows to mount an immune response to *O. ostertagi* [54, 55]. In addition, the uneven distribution of GINs among hosts may reflect the varying grazing behaviours [56]. It should be noted that the statistical model in this study relates inherently correlated values (ODR in individuals vs. ODR in a pooled sample), in addition to the use of an exposure variable on population level, while the outcome is on the individual level. The *a priori* correlation between these measurements of antibodies was not accounted for in the analysis. There is thus a potential bias in assuming causal association at an individual level.

The use of FEC in adult cows is generally not considered useful, and the low fraction of adult faecal samples with a positive EPG is similar to results from other studies [24, 57]. The lack of correlation between log EPG and IS ODR values has also previously been reported in calves [58]. Furthermore, a poor sensitivity and high variability using the McMaster technique at low egg count levels is recognised [59], and in our study herds, this might have caused an underestimation of the FEC in some samples.

The findings in this study are similar to those from previous research, which support the use of individual sampling to assess exposure [51] or the inclusion of both individual and BTM tests in a monitoring programme [30], rather than relying on BTM tests. On the other hand, testing individual animals is time consuming and less practical than testing a single BTM sample per herd, and may not be feasible or economically sustainable in many dairy herds. Another option, not explored in the current study, could be repeated BTM-testing, as a more robust approach than relying on a single BTM sample. The potential value of this would need to be assessed in further studies. As collection and testing of BTM is performed routinely, the result of the *Ostertagia* test could easily be incorporated as part of the herd-health surveillance programme. Moreover, when plotting the IS/IM ODR results according to four BTM categories, the finding of differing ODR values indicates that the evaluation of the BTM test to assess exposure to GIN could be useful in herds with very high or very low BTM ODR values. However, as there were only five herds in our study that are in these categories, we recommend a cautious approach to making inferences about this.

The current study was performed using data from a single sampling occasion and relatively few farms compared with other studies [25, 30, 52]. The small sample size is an important limitation and means that the results are less conclusive results than if more herds had been included. In order to obtain enough samples from FSG, it was necessary to select herds that were larger than the average Norwegian milk production unit, which may have caused a selection bias. The antibody titre could have been influenced by the fact that the average milk production in the studied herds was slightly higher than that of the typical Norwegian dairy farms. Herds were also selected based on a likely greater exposure to GINs, meaning the ODR values in our study might be overall higher than in the target population, and the presented results should not be interpreted as estimates of seroprevalence. The possible impact on the ODR results due to cross-reactions of the ELISA test antigen with *F. hepatica* may have affected the results. Conversely, cross-reactions with *C. oncophora* is not deemed to be a disadvantage, since the ELISA may be considered as an estimation of total GIN exposure [3]. The inclusion of one organic herd was not considered to affect the results, as the probability of parasite exposure was the same in all herds in the study regardless of the production system. Despite the included herds covering the scale from low to high BTM ODR values, the study would have benefited from a larger sample size for better coverage of low and high ODR herds. A future screening of many herds could be one approach to obtaining new knowledge of prevalence and associations between *O. ostertagi* antibodies and herd characteristics.

## Conclusions

There was high variability between the within-herd individual ODR values of both serum and milk in every herd. Although poor predictive ability for BTM ODR to assess individual ODR values in both FSG and cows was demonstrated, the BTM test might be useful to differentiate between levels of GIN exposure in herds with high and low ODR value in BTM. Future studies should include more herds with low and high antibody levels to further evaluate the use of BTM in these herds. This work provides knowledge to guide further studies on GIN, including prevalence studies, investigation of effect on production impact, and risk-factor analysis.

## Data Availability

The datasets used and analyzed during the current study are available from the corresponding author on reasonable request.

## Declarations

### Prior publication

Data included in this article have previously been published in the Proceedings of the 16th International Symposium on Veterinary Epidemiology and Economics Congress, Halifax, Canada, August 7th - 12th 2022.

## Abbreviations

BTM: Bulk tank milk
DIM: Days in milk
ECM: Energy corrected milk
ELISA: Enzyme linked immune sorbent assay
EPG: Eggs per gram
FEC: Faecal egg count
FSG: First season grazers
GIN: Gastrointestinal nematodes
IM: Individual milk
IS: Individual serum
ODR: Optical density ratio
